# Supplementary material for: Are carbon emissions trading and green financial instruments synergistic? -Comprehensive quantitative research based on content analysis
Source: PLoS One. 2024 Mar 7;19(3):e0298601. doi: 10.1371/journal.pone.0298601 (PMC10919601; doi:10.1371/journal.pone.0298601)
Supplement: S1 Data — (ZIP) [file pone.0298601.s001.zip › Data/地级市Z_F工作报告.zip!/╜¡╬≈/╔╧╚─╩╨/└┤╫╘╣1⁄2╓┌║┼í╛┬φ┐╦╩2╛▌═°í┐_2017.docx]

各位代表：

现在，我代表市人民政府向大会报告工作，请予审议，并请各位政协委员和列席会议的同志提出意见。

一、2016年工作回顾

过去一年，是实施“十三五”规划的第一年，也是市、县、乡三级换届之年。面对复杂严峻的经济形势和艰巨繁重的发展任务，在省委、省政府和市委的坚强领导下，在市人大、市政协的监督支持下，我们团结和依靠全市人民，积极适应经济发展新常态，深入贯彻落实新发展理念，攻坚克难，奋力拼搏，较好完成了市三届人大七次会议确定的目标任务，顺利实现了“十三五”的良好开局。全市生产总值1811.1亿元，增长9%。财政总收入302亿元，同口径增长9.2%，其中一般公共预算收入227.5亿元，同口径增长9.1%。固定资产投资1790.3亿元，增长14.7%。规模以上工业增加值751.2亿元，增长9.3%。社会消费品零售总额721.1亿元，增长12.6%。城镇居民人均可支配收入29153元，增长8.3%。农村居民人均可支配收入11103元，增长9.8%。主要经济指标增幅全部达到或超过全省平均水平。

一年来，我们主要做了以下五个方面的工作：

（一）加大投资力度，项目推进富有成效

过去一年，我们把项目作为经济工作的“牛鼻子”，锁定目标、倒排时间，细化任务、狠抓督查，项目建设为稳增长提供了有力支撑。

项目谋划成效明显。结合市“十三五”规划编制，谋划和申报了一批事关全市经济社会发展全局的重大项目。昌景黄高铁已做好前期准备工作，鄱阳至余干快速公路项目列入全国交通扶贫“双百”工程，鄱阳港项目列入省“十三五”规划，沪昆高速公路拓宽升级、信江高等级航道列入省“十三五”重点交通建设项目。上饶动车组存车场项目获得中国铁路总公司批复同意，坑口铁路专用线已与中国铁路总公司签订投资协议，上饶国际物流园已取得立项备案，上饶城市轨道交通项目初设方案、城东旅游综合体项目规划方案基本确定。把握国家政策的“窗口期”，争取专项建设基金80亿元、政府置换债券88.5亿元，为项目谋划和推进提供了资金保障。

项目建设进度加快。三清山机场基本建成并试飞成功。上万高速公路、320国道上饶城区段改建、上丰大道建成通车。九景衢铁路上饶段完成铺轨。德兴潭埠、上饶东两个500千伏变电站投付使用。大坳引水工程建成通水。市群众艺术馆、市精神卫生中心建成并投入使用，市人民医院儿科住院大楼已经封顶，市博物馆、市规划展示馆（城建档案馆）已完成主体框架。上饶大道南二段、明叔大道南段、吴楚大道、稼轩大道、庆丰北路、葛仙山路等城区路网项目建成通车。城东防洪堤、三江导托渠，市人民医院城北院区、市妇幼保健院、市第四幼儿园及上饶大道北段、天佑大道、龙潭路等项目按序时进度推进。

项目督查力度空前。针对全市100个重大项目和省、市《政府工作报告》确定的89个重点项目，分别建立督查微信群，分类成立项目督查组，实行“每周实地督查、每月形象通报”，进一步压实了工作责任、提高了推进效率。通过强力督查推进，14个列入省重点工程的项目完成投资42.5亿元，74个省调度的大中型项目完成投资287.6亿元，均超额完成年度计划；市政府重点调度的89个项目中，计划竣工的21个项目已竣工19个。

（二）推进产业升级，发展质量明显提升

过去一年，我们以“产业兴市”为核心，坚持“主攻工业”战略不动摇，协同推进现代农业和现代服务业发展，产业经济成为稳增长的主动力。

工业集聚能力明显增强。确立了“五年决战七千亿”战略目标，把光伏、光学、汽车“两光一车”产业作为主攻方向。晶科能源四期项目建成投产，成为全球最大的光伏组件制造商。上饶经开区光学产业基地入驻企业39家，其中20家已投产，成为全国三大光学加工基地之一。以汉腾汽车、中汽瑞华、博能客车、爱驰亿维等整车项目为牵引，上饶经开区已落户汽车及配套项目36个。汉腾汽车用四个月在全国开设4S店182家、销售2.4万辆，腾勒汽车发动机项目实现量产，博能客车年销量突破2000辆。2016年，全市新增规模以上工业企业240户（总数达1162户），规上工业企业主营业务收入达到3300.2亿元，增长9%，规上企业数和主营业务收入在全省的排位均比上年前移一位。

农业现代化进程继续加快。着力转变农业发展方式，推动农业基础优势转化为经济发展优势。粮食总产量稳定在70亿斤以上，农产品出口总额和水产养殖面积、产值、产量均列全省第一。累计投入112.3亿元，打造现代农业示范园区12个、特色产业园49个，园区实现总产值189亿元。全市471家市级以上农业龙头企业实现销售收入322.9亿元，带动农民123.9万户。

服务业发展提速增效。全市服务业增加值711.7亿元，占GDP比重达到39.3%，较上年提高1.5个百分点。新增省级现代服务业集聚区5个、省级服务业龙头企业5家。积极开展“国家全域旅游示范区”创建工作，弋阳龟峰获批国家5A级景区，新增4A级景区5个，全市4A级以上景区达到25个，列全省第一；上饶旅游集团景区股权合作及旅游资产并购工作加快推进，第四届“万里茶道”中蒙俄市长峰会、第三届“金驹杯”世界大学生摄影展成功举办；2016年，全市接待游客1.2亿人次、旅游总收入1126亿元、门票收入18.2亿元。加快发展商贸服务业，万达广场、万力时代、明珠商业广场等大型城市综合体建成开业，全市电子商务交易额487.5亿元，增长50.3%。大力发展金融业，新开业商业银行市级分行2家、村镇银行2家、保险公司市级机构3家、证券营业部3家，农信社改制组建农商行全面完成；银行业金融机构存、贷款余额分别为2716.2亿元、1742.1亿元，增长20.3%、18.2%。积极发展大数据产业，高标准规划2000亩大数据产业园，引进大数据企业11家，华为江西云计算数据中心、江西省旅游大数据中心落户上饶，与中科院合作设立的大数据研究院成为全省首个创新型研发机构。

（三）坚持建管并重，城乡面貌不断改善

过去一年，我们以新型城镇化为引领，统筹城乡协调发展，下大力气完善基础设施，多措并举改善城乡面貌，人民生活的家园更加美丽宜居。

城镇化建设深入推进。落实“一核两轴两板块”城镇化空间格局，调整完善土地利用总体规划，报批新增建设用地3万亩，供地5.7万亩，消化“批而未用”土地1.3万亩。320国道各县域段升级改造已确定实施方案，信江河谷板块交通条件进一步改善。统筹推进户籍制度改革，完成农业户口改登30.1万人。在全面完成省下达2.5万户各类棚户区改造任务的基础上，制定了涵盖6.8万户、1300万平方米的城市棚改“三年行动计划”，已完成征迁1.9万户、472.4万平方米。

中心城区面貌明显改观。完成了高铁新区、地下综合管廊、城市景观风貌等规划编制，基本完成城市总规修编。上丰大道加速了广丰融入主城区步伐，中心城区建成区面积达到112.5平方公里、常住人口91.2万人。中心城区出租车行业改革顺利推进，434辆新车全面运营；新增公交线路9条（总数达36条）。完成上饶东、西两个高速公路出口景观改造，启动城区市容网格化管理考核，改造提升公园绿地6万平方米，新增城市亮化楼宇110栋，消除城区道路积水点16处，重建（改造）公厕23座。

秀美乡村建设步伐加快。改造硬化农村公路1800公里，新（改）建桥梁45座，实施水利项目69个。筹措资金14.9亿元，建设新农村点1394个，惠及农民8.95万户。加快网络基础设施建设，光纤宽带实现“村村通”。落实“限高、限大、治乱”要求，进一步规范了农村建房管理。投入农村环境卫生治理资金4.7亿元，农村生活垃圾有效处理率达到82%。大力开展高铁沿线环境整治，依法拆除违章建筑6.7万平方米，复绿山体1140亩，改造房屋外立面7303栋，为打造“中国最美高铁线”作出了“上饶贡献”。

生态文明建设扎实推进。植树造林26.2万亩，淘汰黄标车及老旧车8605辆，均超额完成省下达任务。扎实推进大气环境联防联控，城市空气质量优良率90.4%（优良天数331天）。顺利完成G20峰会“西湖蓝”行动保障任务。深入落实“河长制”，主要河流断面水质达标率稳定在95%以上。新建污水管网46.5公里，全市12座城市污水处理厂基本稳定运行，日均处理量21.1万吨。完成16个全省百强中心镇污水站建设年度任务，建成管网82公里。婺源县与乐平市合作实施了全省首个生态横向补偿试点。全市新创建省级生态乡镇11个、生态村9个，均列全省第一。

（四）深化改革开放，发展活力有效释放

过去一年，我们聚焦向改革开放要动力，坚定不移推进改革攻坚，千方百计促进创新创业，有效拓展了经济社会发展新空间。

深化改革力度加大。全面完成去产能年度任务，关闭煤矿44处，压缩产能192万吨；着力化解房地产库存，去化时间由24个月压缩至7.5个月；落实“降成本、优环境”政策措施，为企业减负40.3亿元。深化财税体制改革，全面推开“营改增”，健全国地税合作机制，通过综合治税平台查补税款及滞纳金13.4亿元。上饶高铁经济试验区管委会正式设立。上饶国际医疗旅游先行区获省政府批复，成为全国第3个医疗旅游先行区。成立市交通投资集团、绿色产业投资集团和上饶供销集团，以上投集团为龙头的“1+7”国有投融资公司体系基本形成。党政机关公车改革顺利完成。加快推进农村土地“三权分置”、集体产权制度、农业保险等改革，农村土地承包经营权确权登记颁证工作位居全省前列。强化质量管理，设立“市长质量奖”，代表江西接受并顺利通过国务院质量工作考核。

扩大开放硕果累累。坚定不移推进产业招商，坚持“一把手”招商和“小分队”招商相结合，洽谈推进项目676个。持续推进“招大引强”和“饶商回归”，签约10亿元以上重大项目57个，开工建设的高铁经济试验区10大项目中，饶商总部基地、建筑科技产业园、电竞总部基地、农产品交易冷链物流园等4个项目均由饶商投资。扎实推进农业招商，引进农业项目18个，总投资21.9亿元。一年来，引进省外资金、利用外资、外贸出口分别为576.7亿元、10.4亿美元、38.4亿美元，分别增长13.3%、10.4%、0.6%。

创新创业氛围浓厚。广丰经开区、弋阳工业园区获批省级高新技术产业园区。全市新增高新技术企业50家（总数达100家），新建市级院士工作站5个，新增省级工程技术研究中心4个，新增省级协同创新体2个，获批省级科技企业孵化器1个、省级“众创空间”8个。全市高新技术产业增加值占工业增加值的比重28.9%，较上年提高3.3个百分点。市、县、乡、村四级就业创业服务网络基本建成，完成旅游、电商、农家乐等创业培训2.2万人次。新增创业担保贷款16.1亿元，直接扶持创业8794人，带动就业3.9万人。德兴市被列入全国首批农民工返乡创业试点单位。全面推行企业“五证合一”和个体工商户“两证合一”登记制度，新增私营企业1.3万户、个体工商户3.3万户，分别增长31.9%、14.6%。全市非公有制经济增加值1122亿元，增长9.9%。

（五）回应群众期待，民生福祉继续增进

过去一年，我们牢记宗旨，全力推进脱贫攻坚，认真办好民生实事，让人民群众有了更多的获得感、幸福感。

脱贫攻坚稳步推进。实施产业扶贫项目575个，惠及贫困户2.6万户，户均增收2476元；争取光伏扶贫电站建设规模260兆瓦，占全省的48%； 126个村被列入“全国乡村旅游扶贫重点村”；落实易地扶贫搬迁对象2748户、1.2万人，为省下达任务的104.6%，建成集中安置点62个。加大财政资金整合和融资对接力度，实施贫困村村庄整治项目2704个。一年来，全市退出省级贫困村112个、实现脱贫13.7万人，均超额完成年度任务。

民生保障不断完善。扶持就业创业，组织各类招聘活动340场次，新增城镇就业5.96万人，新增转移农村劳动力9.6万人，分别完成年度任务的126.2%、132.8%。整合城乡居民基本医疗保险制度，完成城镇居民基本医疗保险和大病保险政策调整，每人筹资标准分别提高40元、15元。提高困难群众生活保障水平，城乡低保和农村五保供养保障标准月提高30元以上。上饶被列入全国15个长期护理保险制度试点城市。筹措资金3亿元，支持农村危房改造2.5万户。

社会事业繁荣发展。大力推进义务教育均衡发展，第二批4个县（区）全部通过国家义务教育均衡发展督导评估。着力缓解“大班额、大校额”问题，中心城区规划新建或改（扩）建学校13所，全部建成后将新增学位3万个。“腾讯智慧校园”全国唯一试点项目落户上饶县。深化医药卫生体制改革，开展城市公立医院改革试点，医疗费用个人负担比例下降2个百分点。落实“全面两孩”政策，计生服务管理水平得到提升。完善公共文化服务体系，新增影院5家（总数达42家），实施文化遗产保护项目45个，广播电视“户户通”工程全面完成。顺利举办2016斯诺克世界公开赛、中华龙舟大赛、第七届环鄱阳湖国际自行车大赛、婺源和上饶国际马拉松赛等体育赛事。

社会治理得到加强。上饶市被评为全国“六五”普法先进城市、全国防震减灾先进单位、全国双拥模范城。科学处置广丰区“1·20”烟花爆燃事故，大规模、高频率开展安全隐患排查，进一步促进了安全生产责任落实。着力提升食品药品检验检测能力，全市食品药品安全形势平稳向好。严厉打击严重违法犯罪行为，现行命案全部告破，成功侦办“重金求子”诈骗案，该案被公安部列为打击电信诈骗的经典案例。加大矛盾问题化解力度，初信初访办结率、信访事项息访率分别为100%、85%。加强应急管理，尤其是面对昌江流域超20年一遇特大洪水及鄱阳湖持续一个月超警戒水位，全市上下军民团结、众志成城，取得了抗洪抢险救灾的全面胜利。

此外，统计、审计、工会、妇女儿童、民族宗教、档案、保密、农垦、人民防空、国家安全、涉台事务、民兵预备役、国防动员、气象水文、检验检疫、无线电管理、住房公积金、机关事务、社科研究、地方志、邮政、通讯、石油、烟草、盐业、老龄、关心下一代、红十字、残疾人、慈善等事业都取得新成绩。

一年来，我们着力加强政府自身建设，转变政府职能，改进工作作风，政府工作效率和服务水平得到提高。深入推进法治政府建设，健全并落实常务会议学法、政府法律顾问列席等制度，严格依法行政，办理行政复议案件66件。自觉接受市人大及其常委会的法律监督、工作监督和市政协的民主监督，办理人大代表建议82件、政协提案190件，按时办复率100%、满意率91.9%、基本满意率8.1%。加强政府及部门网站建设，公开信息2.9万条；“市长热线”收集群众诉求4.8万个，办结率100%、满意率94.5%。扎实开展“两学一做”学习教育，认真履行“一岗双责”，深入开展“集中整治和查处基层侵害群众利益不正之风和腐败问题”“违规插手工程项目”等专项治理，立案528件，处理1908人。

各位代表，过去一年的成绩和进步来之不易，这是省委、省政府和市委坚强领导的结果，是全市广大干部群众积极进取、奋力拼搏的结果，也是社会各界人士积极参与、共同努力的结果。在此，我代表市人民政府，向全市人民，向人大代表、政协委员，向各民主党派、工商联、无党派人士和各人民团体，向驻饶部队、武警官兵、公安民警和中央及省驻饶单位，向广大老干部、老同志，向广大投资者和所有关心支持上饶经济社会发展的同志们、朋友们，表示衷心的感谢！

需要向代表说明的是，受经济下行压力较大等因素影响，投资、出口、居民收入增幅略低于预期目标。同时，我们也清醒地看到，经济社会发展还面临不少困难和问题。主要是：经济总量偏小，人均水平较低，经济整体实力不强，发展不足仍然是当前面临的主要矛盾；产业结构有待进一步优化，实体经济面临较多困难，工业的支柱地位和拉动作用仍不明显；城乡建设管理尚存“短板”，教育、医疗等公共服务供给水平与群众期待还有不小差距；少数政府工作人员的服务意识、担当精神和执行能力还有待进一步提高。对此，我们将积极面对并认真加以解决。

二、2017年主要工作

今年是党的十九大召开之年，也是全面落实市第四次党代会决策部署的第一年，做好政府各项工作至关重要。政府工作的总体要求是：全面贯彻党中央、国务院、省委、省政府和市委的总体部署，坚持稳中求进工作总基调，牢固树立和贯彻落实新发展理念，主动适应经济发展新常态，强化“六种意识”，加快“七区”建设，全面做好稳增长、促改革、调结构、惠民生、防风险各项工作，奋力迈出决胜全面小康、打造大美上饶的坚实步伐，以优异成绩迎接党的十九大胜利召开！

全市经济社会发展的主要预期目标是：生产总值增长9%左右，财政总收入增长5%左右，规模以上工业增加值增长9%左右，固定资产投资增长14%左右，社会消费品零售总额增长12%左右，实际利用外资增长9%左右，外贸出口增长1%左右，城镇居民人均可支配收入增长8.5%左右，农村居民人均可支配收入增长9.5%左右，居民消费价格总水平涨幅控制在3%左右，城镇登记失业率控制在4%以内，节能减排完成省政府下达的计划任务。

实现上述目标，我们将重点抓好以下六个方面的工作：

（一）加快构建特色鲜明的工业体系

发展壮大主导产业。围绕“五年决战七千亿”战略，编制信江河谷城镇群与滨湖板块产业发展规划，市本级瞄准“世界光伏城、中国光学城、江西汽车城”的目标，以大项目和龙头企业为引领，重点发展“两光一车”产业；各县（市、区）重点发展1—2个主导产业，形成优势互补、错位发展的产业格局。积极引进和培育新技术、新产业、新业态、新模式，以新经济引领传统产业转型升级，带动战略性新兴产业发展。抓好汉腾汽车整车二期、彩诚汽车零部件、中汽瑞华新能源整车及零部件、众光照明光电产业基地、广丰卷烟厂易地技改等重大项目建设，生成包装一批延链、补链、壮链的产业项目。

提高园区集聚能力。按照“做强工业园区”的要求，探索实行“决策机构一元化、管理机构扁平化、服务机构企业化”的模式。引导园区提高投入产出率、资源利用率和实际开发度，促进园区集约发展。积极推进产城融合，不断完善园区基础设施和生产生活配套，健全社会化服务体系。全力支持上饶经开区、上饶高新区做大做强，在项目引进、用地、审批等方面优先保障；探索建立上饶经开区与有关县（市、区）合作机制，积极打造协同共建、利益共享的“飞地园区”。

加大企业帮扶力度。坚持“重点工业项目每季度集中开（竣）工”“市县主要领导每月工业日”等工作机制，加大“精准扶企”力度，通过“一企一策”“一事一议”，提高帮扶企业的针对性和实效性。深化“降成本、优环境”专项行动，实施“质量强市”战略，发挥中小微企业转贷互助帮扶基金作用，持之以恒帮助企业降成本、提质量、增效益。通过财政引导、市场化运作，建立“1+N”产业引导基金，以股权投资方式撬动工业转型升级。抓好达规工业企业“入统”工作，力争全市新增规模以上工业企业200家，规上工业主营业务收入达到3600亿元。发挥上饶创业大学作用，继续办好“千名企业家走进浙大”培训班。

（二）加快推进现代农业发展

促进农业融合发展。抓好农业供给侧结构性改革，延伸农业产业链，提高农产品加工转化率和附加值。优化特色产业布局，打造“东柚、西蟹、南红、北绿、中菜”等特色产业集群。抓好农产品仓储物流、批发市场等设施建设，推广农超、农企产销对接，鼓励在城区设立鲜活农产品直销网点。依托田园风光、乡土文化等资源，发展农业新业态，推进农业与旅游、文化、健康、养老等产业深度融合，积极创建国家现代农业庄园。综合运用“互联网+”“旅游+”等手段，做实、做优各地休闲农业综合体，打造成为游客的乐园、市民的公园和农民增收致富的重要平台。

夯实现代农业基础。落实“藏粮于地、藏粮于技”战略，大力推进高标准农田建设，深入开展粮食绿色高产高效创建。加强农田水利设施建设，实施水利项目445个，有效提升灌溉保障水平和防汛抗洪能力；加快推进城东防洪堤、三江导托渠等项目建设，开工建设马鞍山水利枢纽、万年夏营水库等项目。推进农产品标准化生产、品牌创建、质量安全监管，增加绿色有机优质农产品供给。继续健全农业科技推广体系，提高农业科技创新能力。

（三）加快发展高品质服务业

积极落实大数据产业布局。按照“做热高铁经济试验区”的要求，围绕“一个园区、八个中心、一个基地”的建设思路，打造江西省数字经济示范区。加快推进一呼百应、健康之路、迈普医学、数联铭品等项目落地运营，继续加强大数据产业招商，形成集“基础设施、应用平台、外包服务”于一体的大数据产业链。积极探索“互联网+特色小镇”模式，按照“一村一业”模式建设数字小镇，努力将其打造成为“机制灵活、业态多样”的创新创业空间。加强与知名高校、院所和企业合作，引导一批高层次人才来饶创新创业，不断提高大数据产业化、市场化水平。

创建国家全域旅游示范区。按照“做旺全域景区”的要求，抓好重点景区拓展升级，强化景区全域化合作，缓解景区景点“业态单一、游程较短”和“冷热不均、淡旺季明显”等问题。大力发展智慧旅游，继续推进“厕所革命”，规范旅游市场管理，不断提升旅游服务质量。开工建设城东旅游综合体、信江夜游等项目，提升中心城区旅游吸引力。推动婺源篁岭等条件较好、积极性较高的地方创建国家5A级景区，支持玉山怀玉山、铅山葛仙山、余干大明湖创建国家4A级景区，努力实现“县县有4A级景区”的目标。按照“做优国际医疗旅游先行区”的要求，抓好干细胞再生医学、辅助生殖、基因排序检测等项目；大力发展中医药产业，积极争创国家中医药健康旅游示范区。力争全年旅游总收入突破1300亿元，门票收入突破21亿元。

大力繁荣商贸物流业。推动商贸业线上线下融合发展，深入推进“电子商务进农村”，启动实施“电子商务进社区”，大力发展跨境电商，积极争创国家级电子商务示范基地。实施城乡菜市场改造提升工程，打造示范菜市场10个。按照“做活物流新区”的要求，推广统一配送、共同配送等物流模式，积极争取物流标准化建设试点，开工建设上饶国际物流园，加快推进农产品交易冷链物流园建设。按照“做实空港新区”的要求，统筹推进航空配套、现代物流等服务业发展。

提高金融业服务水平。优化政银企融资对接服务平台，着力解决金融“脱实向虚”问题。加快组建由政府出资控股或参股的融资性担保机构，为中小企业融资提供增信服务，进一步缓解企业融资难问题。充分发挥资本市场服务中心作用，用好国家支持贫困县企业上市等政策机遇，加强企业上市培育工作，扩大直接融资规模。健全信用监督和失信惩戒制度，大力化解不良贷款，防范和打击非法集资活动，不断优化金融生态环境。

（四）加快建设美丽上饶

完善交通配套设施。抓好三清山机场航线开辟等工作，确保5月正式通航。做好九景衢铁路年内建成运行的各项服务保障工作。加大协调力度，力争开工建设坑口铁路专用线、高铁动车组存车场、鄱阳至余干快速公路、信江高等级航道、万年港综合码头二期工程，加快推进320国道各县域段升级改造项目，做好上饶城市轨道交通、沪昆高速公路上饶段拓宽升级等项目前期工作。

做美做靓中心城区。按照“做大中心城区”的要求，完成《上饶市城市总体规划》报批，完成中心城区基础设施规划和海绵城市建设专项规划编制，加快编制各片区控规及城市设计，做好城市建设用地储备工作。优化中心城区基础设施，年内新增城区道路30公里以上，加快推进稼轩大道、明叔大道北延伸和广平街南延伸等项目建设，确保上饶大道、天佑大道全线贯通。大力推进公共停车场建设，着力解决“停车难”问题。统筹地上地下建设，建成地下综合管廊7.3公里，改造雨污水管网50公里，逐步实现弱电下地、雨污分流。加强融资对接，加大征迁力度，全面完成各类棚户区改造任务。进一步加强城市管理工作，完善体制机制，压实各级责任，推进“重心下移”。严格落实属地管理、行业监管和相关部门职责，不断提升住宅小区物业管理水平。深入推进城市建成区违法建设专项治理，推动出台《上饶市城市管理条例》和《上饶市市容环境卫生管理办法》。以申报全国文明城市为契机，抓好精神文明建设，培育文明道德风尚。

在继续推进市档案馆、市美术馆、市老年人综合活动中心等重点项目建设的同时，今年中心城区还要着力办好10件（类）民生实事：一是新建3所学校（市叶挺学校、市带湖学校、市明叔小学），改（扩）建3所学校（市信江学校、市时乔小学、市十五小）。二是新建市委党校新校区。三是新建妇女儿童、青少年、演艺、职工综合活动中心和市老年福利中心。四是新建3个城区公共停车场、2个公交综合服务中心，新购100辆新能源公交车。五是新建3个市场（石材市场、废旧钢材市场、四脚亭集贸市场）。六是改造提升7条城区主干道（凤凰大道、三清山大道、紫阳南大道、五三大道、站前大道、书院路、丰溪路）。七是新建1.7万平方米街头绿地小游园。八是新设1100个街头垃圾箱、30座公厕驿站、500个消火栓。九是新建市安全生产救援基地和救灾物资储备库。十是新建中心城区生活垃圾焚烧发电项目和市餐厨垃圾处置中心。

大力建设秀美乡村。建设农村公路1800公里，新（改）建桥梁50座，确保25户以上自然村水泥路通达率超过90%。全面推进铁路、高速公路、国省道等主要通道沿线环境整治，建设“秀美乡村”村点3000个。深入推进农村环境卫生整治，继续实施农村饮水提升改造工程，重点抓好农村生活垃圾和污水治理，大力整治农业面源污染。完善乡镇规划所职能，持续加强农村建房规划管理，让农村的房子“看起来更舒服、住起来更实用”。培育壮大村级集体经济，提高村级组织公共服务能力和运转保障水平。支持农村特色产业发展，加强文化遗存保护，建设“一村一品、一村一景、一村一韵”的魅力村庄。加强农村精神文明建设，创新乡贤文化，发挥“村规民约”的教化、约束作用，弘扬善行义举，树立文明乡风。

加强生态文明建设。完成植树造林13.7万亩，力争年内成功创建国家森林城市。全面开展第二次污染源普查，建立和完善污染源及治理设施数据库。编制和落实水、大气、土壤污染防治行动计划，对主要河流县（市、区）交接断面实行全面水质监测；继续淘汰燃煤小锅炉和黄标车，全面建成县级环境空气自动监测网络；大力推进国、省控重点防控区重金属污染综合防治，开工建设市工业废弃物综合利用及无害化处置中心。配合做好省以下环保监测执法机构垂直管理改革工作。推动上饶循环经济产业园“一市场四中心”竣工投产，力争顺利通过商务部验收。

（五）加快培育发展新动能

抓好重点领域改革。深入推进“三去一降一补”，有序化解过剩产能，促进房地产市场平稳健康发展，支持符合条件的企业开展市场化、法治化“债转股”。支持建筑业转型升级、做大做强，积极发展装配式建筑。深化“放管服”改革，大力推动行政服务事项网上办理，整合市县公共资源交易平台，不断提高行政服务质量和效率。抓好国资国企改革，推动行业相近、功能类似、业务关联的市属国有企业兼并重组，支持有需求、有条件的国有企业开展混合所有制改革试点。理顺上饶高铁经济试验区管理体制，完善项目引进、落地、推进等服务机制。扎实推进工程招投标体制改革，实行“一委一办一中心”管理模式，实现“决策、执行、监督”相分离。启动事业单位和国有企业公务用车改革。继续深化户籍制度改革，全面完成户口一元化改登工作。巩固农村土地承包经营权确权登记颁证工作成果，深入推进农村集体经济组织产权制度改革试点，抓好婺源县农民住房财产权抵押贷款改革全国试点。加大国有森林资产整合力度，力争上饶绿投集团资产规模达到300亿元。抓好垦区集团化省级试点和农业部深化农垦改革专项试点工作。

持续推进双向开放。以产业招商为核心，健全和完善“一把手”招商、“小分队”招商、以商招商、专业公司招商等机制，不断提高招商引资的精准化、专业化水平。落实抓洽谈签约、抓开工竣工、抓核查巡查、抓分析调度的“四抓”工作机制，切实提高招商项目落地转化率。大力推进饶商回归，办好2017全球饶商回归大会，筹备成立陕西、广西等异地上饶商会。落实《“十三五”促进中部地区崛起规划》，顺应经济梯度转移趋势，积极承接东南沿海地区产业转移。以赣港、赣台经贸活动和厦门投洽会等省办招商活动为契机，举办走进珠三角、闽东南、长三角等系列招商活动。抓好上饶“无水港”搬迁扩建、保税物流中心申报建设、“海铁联运天天班”常态化运行等工作。大力引进和培育生产型出口企业，帮助更多出口企业取得国际市场通行证，年内新增生产型出口企业20家。力争全年引进省外资金、利用外资、外贸出口分别达到635亿元、11.3亿美元、38.8亿美元。

激发创新创业热情。加强科技服务平台和创新载体建设，力争建成省级工程技术研究中心5个、省级“众创空间”5个、省级科技企业孵化器2个，组建省级科技创新团队3个。继续强化企业创新主体地位，力争新增高新技术企业30家以上。支持和鼓励全社会加大研发投入，切实提高R&D经费支出占GDP的比重。继续实施就业促进计划和创业引领计划，开展“订单式”“定向式”职业技能培训，落实创业担保贷款新政策，重点扶持返乡农民工和高校毕业生等重点群体创业。支持和引导上饶师范学院、上饶职业技术学院等与企业和社会各界的深度合作，提高本地院校服务经济社会发展的能力和水平。巩固企业“五证合一”、个体工商户“两证合一”改革成果，进一步降低准入门槛，营造更加宽松的创业环境。

（六）加快全面小康进程

扎实推进精准脱贫。加大资金整合力度，完善贫困地区交通、电力、通讯、农田水利等基础设施，推进425个省级贫困村村庄整治。重点抓好产业扶贫，推广“合作社+贫困户”模式，因地制宜实施一批带动力强、稳定性好的产业项目。有序稳妥实施易地扶贫搬迁安置，确保搬迁户搬得出、能就业、有收入。扎实做好兜底保障脱贫，统筹推进健康、教育等脱贫工作。压实结对帮扶责任，鼓励和引导社会力量参与扶贫、支持扶贫。坚持扶贫、扶志并举，激发贫困群众内生动力，提高自我发展能力。确保完成2个贫困县摘帽，210个省级贫困村、4个市级贫困村退出，14.5万贫困人口脱贫。

完善社会保障体系。健全基层劳动就业和社会保障平台，落实“稳岗补贴、见习补贴、社保补贴”等政策，新增城镇就业4.7万人、转移农村劳动力7.2万人。加快建设“多险合一”信息系统，实现养老、医疗、工伤、生育、失业等社保费统征分付。加大财政保障民生力度，城乡低保保障标准月增加35元以上，农村特困人员供养标准月提高30元以上。大力发展养老服务业，深化养老保险制度改革，抓好长期护理保险制度国家试点。

大力繁荣社会事业。优化学校网点布局，扩充城镇教育资源；全面部署“腾讯智慧校园”建设，提升基础教育信息化水平；促进义务教育均衡发展，力争弋阳、万年、三清山顺利通过国家义务教育均衡发展督导评估；继续支持上饶师范学院创建上饶师范大学、江西医学高等专科学校建院升本；推进上饶幼儿师范高等专科学校校区建设，确保今年秋季在新校区开学。深化医药卫生体制改革，控制医疗费用不合理增长，有效引导“分级诊疗”，提高医疗卫生服务供给效率。加快构建现代公共文化服务体系，重点推进基层综合文化服务中心建设，实施文化创作繁荣工程和地方戏曲振兴工程，办好首届上饶文化创意产业博览会和第十四届四省四市民间艺术节。落实《全民健身实施计划》，办好市第四届运动会，建成市体校项目，创新发展体育服务业。

维护社会安全稳定。全面落实安全生产属地管理职责、部门行业监管职责、企业主体责任，完善风险管控和隐患治理机制，及时消除安全隐患。健全食品药品安全责任体系，提高检测和监管能力，确保人民群众吃得安全、用得放心。扩大“天网”覆盖面，加快建设立体化、信息化社会治安防控体系。认真做好第十届村（居）委会换届选举工作。完善法律援助制度，推动法律援助扩大范围、提高质量。深入开展“信访积案化解攻坚”活动，重点解决城市房屋拆迁、农村土地征用、村组干部作风、环境污染、非法集资等影响社会和谐稳定的突出问题，依法处理信访活动中的违法犯罪行为。

各位代表，落实发展思路，实现美好愿景，必须进一步加强政府自身建设。我们将严格依法行政，强化决策法定程序的刚性约束，着力提高政府工作的法治化、规范化水平，定期向人大报告工作、向政协通报情况，认真办理人大代表建议和政协提案，自觉接受人大、政协、群众和舆论监督。我们将狠抓工作落实，在坚持“现场督查、微信通报”等做法的基础上，建立健全“总巡查、总督查、总协调”工作机制，压实工作责任，强化绩效评估，严肃行政问责，为敢于担当的干部担当，为敢于负责的干部负责，确保各项决策部署落到实处。我们将坚守清廉为政，全面落实党风廉政建设主体责任，严格落实中央“八项规定”精神和国务院“约法三章”，充分发挥巡察和审计监督作用，防控廉政风险，厉行勤俭节约，维护政府清正廉洁的良好形象。

各位代表，新目标指引新征程，新任务呼唤新作为。让我们紧密团结在以习近平同志为核心的党中央周围，在省委、省政府和市委的坚强领导下，担当实干，砥砺前行，为决胜全面小康、打造大美上饶而努力奋斗！
